# Using next‐generation sequencing to detect oral microbiome change following periodontal interventions: A systematic review

**DOI:** 10.1111/odi.13405

**Published:** 2020-05-26

**Authors:** Ya Zhang, Yinliang Qi, Edward C. M. Lo, Colman McGrath, May Lei Mei, Ruoxi Dai

**Affiliations:** ^1^ The Second People’s Hospital of Hefei Hefei Hospital Affiliated to Anhui Medical University Hefei China; ^2^ Key Laboratory of Oral Diseases Research of Anhui Province Stomatological Hospital & College Anhui Medical University Hefei China; ^3^ Department of Dental Public Health Faculty of Dentistry The University of Hong Kong Hong Kong; ^4^ Faculty of Dentistry University of Otago Dunedin New Zealand

**Keywords:** metagenomic, next‐generation sequencing, periodontitis, systematic review

## Abstract

**Objectives:**

This systematic review was to evaluate the change of oral microbiome based on next‐generation sequencing (NGS)‐metagenomic analysis following periodontal interventions among systematically healthy subjects.

**Materials and Methods:**

A structured search strategy consisting of “metagenomics” and “oral diseases” was applied to PubMed, EMBASE, and Web of Science to identify effective papers. The included studies were original studies published in English, using metagenomic approach to analyze the effectiveness of periodontal intervention on oral microbiome among systematically healthy human subjects with periodontitis.

**Results:**

A total of 12 papers were included in this review. Due to the heterogeneity of selected study, quantitative analysis was not performed. The findings as to how alpha diversity changed after interventions were not consistent across studies. Six studies illustrated clear separation of microbial composition between dental plaque samples collected before and after intervention using principal coordinates/component analysis. The most commonly detected genera before intervention were *Porphyromonas*, *Treponema*, *Tannerella*, and *Prevotella*, while *Streptococcus* and *Actinomyces* usually increased and became the dominant genera after intervention. Correlation network analysis revealed that after intervention, the topology of network was different compared to the corresponding pre‐interventional samples.

**Conclusion:**

Existing evidence of metagenomic studies depicts a complex change in oral microbiome after periodontal intervention.

## 1 INTRODUCTION

Periodontitis is one of the most common oral diseases in the world. The global age‐standardized prevalence of severe periodontitis (SP) in 1990 to 2010 was 11.2%, and the incidence was at 0.7% (Kassebaum et al., 2014). It is a main reason for tooth loss, incurring great expense on dental prosthetic treatment (Petersen & Ogawa, 2005). The etiology of periodontitis is complex, which starts from polymicrobial infection caused by intraoral biofilm, and is accompanied by host inflammatory response, leading to severe destruction of periodontal apparatus (connective tissue, alveolar bone, periodontal ligament).

Around 700 species of bacteria have been identified in the oral cavity by culture‐dependent and culture‐independent tests (Paster, Olsen, Aas, & Dewhirst, 2006). The oral bacterial species associated with healthy gingivae consist of Gram‐positive cocci (*S. mutans*, *S. mitis*, *S. sanguis*, *S. oralis*, *Rothiadentocariosa*, *S. epidermidis*), a few Gram‐positive bacilli (*A. viscosus*, *A. Israelis*, *A. gerencseriae*, *C. spp.*), and very few Gram‐negative cocci (*Veillonellaparvula*, *Neisseria* spp.) (Listgarten, 1976). Socransky grouped oral microbes into several groups, in which orange and red complexes are associated with periodontal diseases, for example, *P. gingivalis*, *P. intermedia*, *T. forsythia*, and *T. denticola* (Socransky, Haffajee, Cugini, Smith, & Kent, 1998). Next‐generation sequencing (NGS) technique, also known as high‐throughput sequencing, differs from traditional Sanger sequencing in that it enables us to sequence hundreds of genes at one time and has a deeper coverage of microbial community (Koboldt, Steinberg, Larson, Wilson, & Mardis, 2013). By using NGS technique, we can sequence either 16S rRNA or the whole genome (metagenomics). 16S rRNA exists in all bacteria and archaea and is a phylogenetic marker, which can be utilized to determine taxonomic composition in the oral cavity. The 16S rRNA amplicon sequencing technique is typically based on the amplification of small fragments usually covering one or two hypervariable regions (such as V1‐V3, V4, or V4‐V5 regions) of the 16S rRNA genes of bacteria/archaea (Ju & Zhang, 2015). The DNA sequences of these amplicons are then mapped to a reference 16S sequence database for taxonomic identification and abundance estimation. While the high‐throughput sequencing of 16S rRNA genes can usually profile taxonomic composition at the genus or species level, the whole genome sequencing can potentially provide species‐ or even strain‐level taxonomic resolution in the human microbiome analysis. Moreover, the whole genome sequencing provides more metabolic features and enables us to further understand functional capacities of microbial communities. The shotgun strategy based on NGS identifies the sequence of entire genomes by producing random fragments of DNA (25–500 bp) and assembling them by computers via overlapping ends (Quince, Walker, Simpson, Loman, & Segata, 2017). In modern‐day studies employing NGS, the terms of metagenomics and 16S rRNA are often used interchangeably.

The cornerstone of periodontal disease treatment is mechanical debridement of supra‐ and subgingival plaque and calculus (scaling and root planning, SRP) (Pihlstrom, Michalowicz, & Johnson, 2005). Chemotherapeutic agents (Van Strydonck, Slot, Van der Velden, & Van der Weijden, 2012) and antibiotics (Keestra, Grosjean, Coucke, Quirynen, & Teughels, 2015) can be used in conjunction with SRP. The effectiveness of those interventions has been evaluated largely in clinical setting or by traditional culture methods/specific sequencing (e.g., 16S RNA probe) before. It is insufficient to get a comprehensive picture as how patients with periodontal diseases benefit from those treatments at the level of compositional and metabolic pathway change in oral microbial communities. Since increasingly more studies use NGS to study the effects of periodontal interventions, this systematic review aimed to summarize and elucidate the changes in the compositional profile and metabolic pathways in the microbial community associated with these interventions.

## 2 MATERIALS AND METHODS

### 2.1 Search methods

The search strategy used in this review was based on the search terms, which were related to either “metagenomics” or “oral diseases.” The purpose of using search terms of “oral diseases” was to get broad search results of metagenomic studies in oral diseases, including caries, gingival diseases, and periodontitis. This paper only reports on the findings of the studies on periodontitis. According to the specific requirements in PubMed, EMBASE, and Web of Science (updated until December 2019), search terms were modified accordingly (Appendix S1). In order to expand the scope of the search, manual search was performed on the list of references in relevant papers and some registry of clinical trials (such as *ClinicalTrials.gov* by US NIH).

### 2.2 Selection criteria

The inclusion criteria for selection of papers in this review were as follows: original studies with the implementation of periodontal interventions (chemical agents, mechanical plaque removal, or systemic administration of antibiotics); used NGS‐based metagenomic approach (16S RNA or/and whole genome) before and after the intervention to analyze oral microorganisms; conducted on generally healthy human subjects; and published in English.

### 2.3 Data collection

Two reviewers first independently screened papers based on titles and abstracts according to the above selection criteria to identify potentially eligible papers. The full text of potentially eligible papers was then obtained. Disagreement between the reviewers was resolved through discussion. The key information of each selected paper was recorded, including study design, sample size, intervention, microbial sampling methods, metagenomic analysis methods, and main findings.

### 2.4 Quality assessment

Two investigators assessed the quality of randomized clinical trials (RCTs) and non‐RCTs included in this systematic review, according to the Agency for Healthcare Research & Quality (AHRQ) Evidence‐based Practice Center (EPC) Methods Guide (Viswanathan et al., 2008). This systematic review was written according to PRISMA guidelines checklist (Table S1).

## 3 RESULTS

### 3.1 Search results

**Figure 1:**
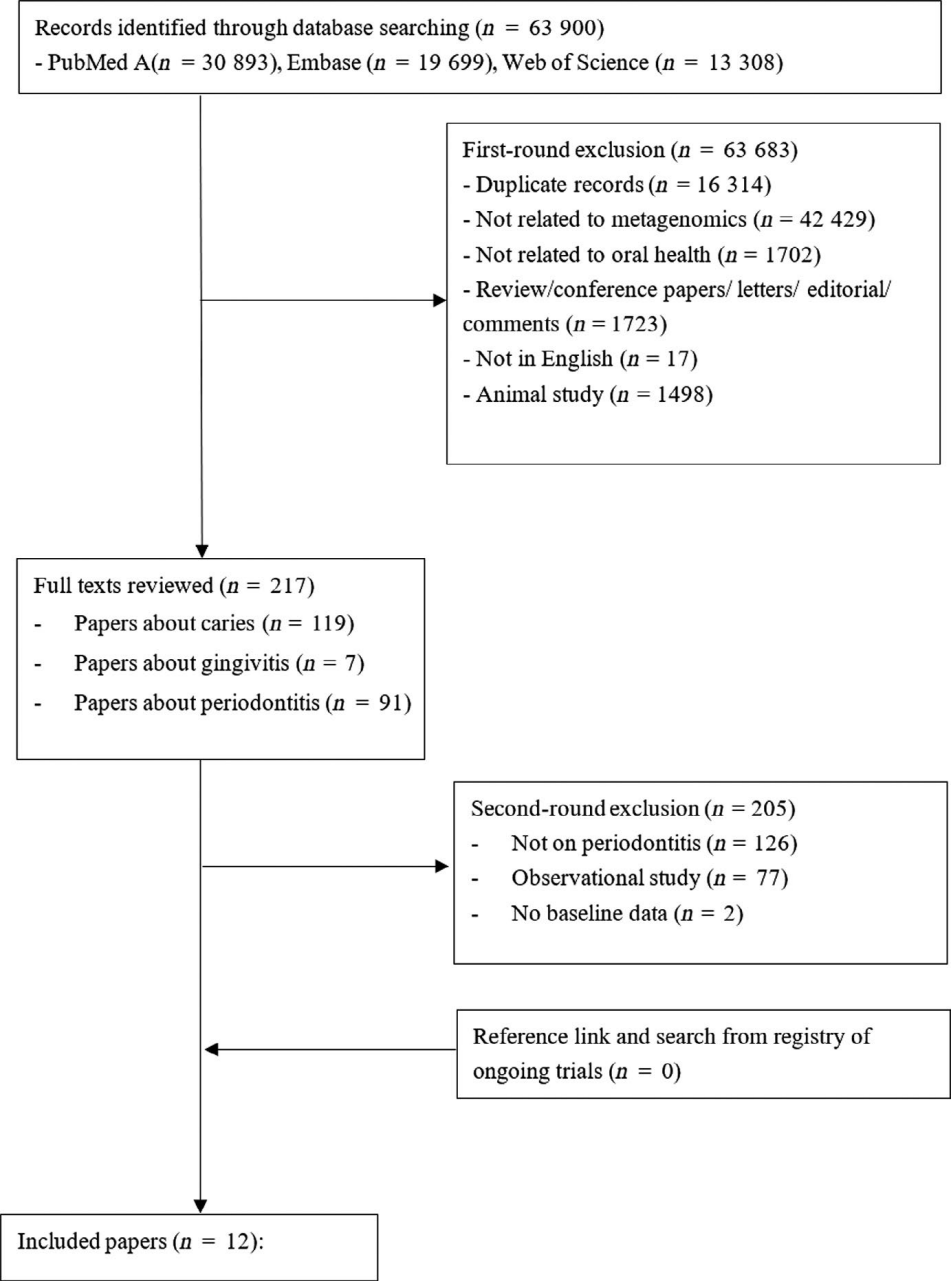
Process of identification and selection of studies for inclusion in review

A total of 63,900 item/papers were retrieved from the three searched databases, including 30,893 articles in PubMed, 19,699 in EMBASE, and 13,308 in Web of Science. After removing duplicates, 47,586 papers were left. In the first round of screening based on titles and abstracts, 42,429 papers not using metagenomic approach, 1,702 papers not related to oral health, 1,723 papers of review/conference proceedings/letters/editorial comments, 1,498 papers of animal studies, and 17 papers not in English were excluded. In the second round of screening based on full text, 91 papers related to periodontitis, 7 related to gingivitis, and 119 related to caries were identified. Among the 91 papers related to periodontitis, 77 observational studies and 2 studies without baseline data were excluded. The remaining 12 papers were included in this review paper (Belstrom et al., 2018; Bizzarro et al., 2016; Califf et al., 2017; Chen et al., 2018; Hagenfeld et al., 2018; Han, Wang, & Ge, 2017; Junemann et al., 2012; Laksmana et al., 2012; Liu et al., 2018; Schwarzberg et al., 2014; Shi et al., 2015; Yamanaka et al., 2012). The screening process is shown in Figure 1. Details of the included studies and summary of their findings can be found in Table 1. The quality of the selected studies can be found in Table S2. The quality assessment showed that the following information is missing commonly in the selected studies: What was the source population the study subjects were selected from, how the confounding factors were adjusted, how the sample size is calculated, and how missing data were handled.

**Table 1:** Summary of the selected studies

Ref no.	Study type	Sample size	Intervention group	Comparison group	Sample type	Sampling method	Metagenomic analysis	Main Findings	Authors’ Conclusion
1	Pre–post interventional study	31 enrolled 25 completed	SRP	NA	Saliva and subgingival plaque	Samples collected at baseline, and 2 weeks, 6 weeks, and 12 weeks after intervention: 1. Chewing‐stimulated saliva sample collected from 8 a.m. to 3 p.m. 2. Subgingival sample collected from four sites with the deepest pocket with curette (pooled, one sample per subject at one time point)	1. Relative abundance: percentage of read counts 2. Alpha diversity	1. Relative abundance of Streptococcus, Rothia, and Actinomyces genera significantly increased, while relative abundance of Porphyromonas and Treponema species significantly decreased as a result of SRP while relative abundance of predominant bacterial genera in saliva remained similar after SRP. 2. Both the alpha diversity of subgingival plaque and saliva significantly decreased after 2 weeks and 6 weeks, and completely reversed after 12 weeks. 3. There was an overall significant correlation between relative abundance of putative periodontitis‐associated pathogens in subgingival and saliva samples at baseline and each postinterventional time point.	SRP had greater impact in decreasing relative abundance of periodontitis‐associated genera in the subgingival plaque than in saliva. Significant correlation of putative periodontitis‐associated pathogens between subgingival and saliva samples indicated that those specific pathogens in saliva samples could reflect also subgingival colonization.
2	RCT	37 Test: 18 Control: 19	0.12% chlorhexidine rinse (twice daily for 28 days) and antibiotics (amoxicillin 375 mg + metronidazole 250 mg) 3 times daily for 7 days	0.12% chlorhexidine	Subgingival plaque	Samples collected from the deepest non‐furcated site in each quadrant (pooled) at baseline, and 3, 6, and 12 months after treatment with paper point	1. Alpha diversity: Shannon index 2. Beta diversity: PCA based on Bray–Curtis 3. Relative abundance 4. Correlation network	1. Between‐group comparison showed that there was no difference in alpha diversity between two groups at any time point. Within‐group comparison showed that there was no significant difference in alpha diversity over time. 2. PCA showed there was only significant difference between intervention and control groups at the 3‐month follow‐up. Within group, there was a difference between pre‐ and postinterventional samples at baseline and all follow‐up periods. 3. Relative abundance showed that *Neisseria*, *Rothia*, *Capnocytophaga*, *Streptococcus* increased and *Filifactor*, *Tannerella*, *Fusobacterium*, *Porphyromonas*, *Treponema*, *Syntrophomonas* in both groups. 4. Among patients who did not respond well to interventions clinically, the topology of correlation network after intervention was similar to the one at baseline, with disease‐associated genera highly interconnected. Among patients who responded well to interventions clinically, the topology of correlation network included a fully connected network of health‐associated genera and four networks of disease‐associated genera at baseline, and changed to one network consisting of one subnetwork of health‐associated genera and one subnetwork of disease‐associated genera.	The change of bacteria composition structure was correlated with intervention and clinical treatment results. The change of alpha diversity and co‐occurrence of specific microorganisms can be useful in predicting the resolution of diseased sites after intervention.
3	Interventional study with concurrent comparison group (randomization not mentioned)	34 Test: 17 Control: 17	0.25% sodium hypochlorite rinse	Water rinse	Supra‐ and subgingival plaque	Samples collected with a sterile Gracey curette at baseline, and 2 weeks and 3 months after interventions: 1. Supragingival plaque (one sample pooled from three teeth per subjects) 2. Subgingival plaque (three samples from three teeth with deep pocket per subjects, not pooled)	1. Alpha diversity: Faith's phylogenetic diversity 2. Richness: Chao1 3. Beta diversity: PCoA based on data of weighted UniFrac and Bray–Curtis 4. Relative abundance 5. Community instability (CIS) analysis (based on data of weighted UniFrac, Bray–Curtis)	1. Higher diversity and number of metabolites were both significantly positively correlated with the maximum pocket depth (MPD). 2. PCoA showed that there was no clear compositional separation of microbiomes across different groups of disease severity in any set of metagenomic data. 3. There was a change in the taxa correlated with MPD among the top 20 relative abundant taxa after intervention compared to baseline (based on both 16S rRNA and shotgun metagenomics). The change was even more significant in metabolites. 4. Greater instability was found in periodontal pockets that did not improve compared to pockets that improved from the data of 16S rRNA, while the finding is the opposite from the data of metabolic features	1. Higher alpha diversity and metabolites were indicators of deep pockets. 2. No periodontitis‐associated pathogen group was found based on beta diversity analysis. 3. Metabolic dynamism was more indicative of the effectiveness of treatment than shifts in composition in community. 4. The positive correlation between taxonomic instability and diseases status further proved the polymicrobial etiology of periodontal diseases, and the negative correlation between metabolites and diseases status may reflect the postinterventional metabolites level of host rather than that of bacterial community.
4	Pre–post interventional study	48 chronic periodontitis 21 healthy at baseline 19 with chronic periodontitis resampled after intervention No healthy subjects resampled after intervention	SRP	NA	Saliva and subgingival plaque	Unstimulated whole salivary samples at baseline Subgingival samples collected from two contralateral maxillary posterior teeth with sterile paper points at baseline and 4 weeks after intervention (not pooled)	1. Alpha diversity: Shannon index, Simpson index 2. Evenness: Pielou's index 3. Beta diversity: PCoA, hierarchical clustering 4. Relative abundance (also illustrated in heatmap) 5. Network analysis (random matrix theory) 6. Community ecological process	1. Alpha diversity was similar among all types of plaque samples, while richness and evenness were significantly less in saliva samples of healthy subjects compared to subjects with chronic periodontitis (both pre‐ and postintervention). 2. PCoA revealed a clear separation between plaque and saliva samples. Heatmap showed disease‐ and health‐associated taxa. 3. Relative abundance showed that 24 taxa, including periodontitis‐associated bacteria, decreased after treatment in plaque samples. Six taxa, part of which were disease‐associated taxa, decreased after treatment in saliva samples. 4. Network analysis showed that the topology of the post‐treatment samples (both saliva and plaque samples) was different compared to the corresponding pre‐intervention samples. Less correlation was found in plaque samples after intervention, while more correlation was found in saliva samples after intervention. 5. Undominated and homogenizing dispersal were the two major factors that dictated bacterial community turnover after treatment.	Microbiota were distinct between saliva and subgingival plaque sample, as well as between disease and healthy sites. SRP was effective in decreasing relative abundance of periodontitis‐associated genera in the subgingival plaque and saliva samples, and changing correlation between bacteria in both subgingival plaque and saliva.
5	RCT	96 enrolled 89 completed Test: 47 Control: 42	SRP + 500 mg amoxicillin + 400 mg metronidazole, three times daily for 7 days	SRP	Subgingival plaque	Samples collected with sterile paper point from four teeth with pocket depth > 6 mm (pooled) at baseline and 2 months after interventions with one sterile paper point	1. Richness: number of ribosomal sequence variants (RSV) 2. Evenness: Pielou's index 3. Alpha diversity: Shannon index 4. Beta diversity: PCoA based on Bray–Curtis 5. Relative abundance: mean read counts	1. Within the group of SRP plus placebo, there were no significant differences in richness, evenness, and alpha diversity between baseline and after intervention. 2. Within the group of SRP plus antibiotics, there were no significant differences in evenness and diversity between baseline and after intervention; however, richness decreased and dissimilarity increased significantly after intervention. 3. PCoA showed that there was a clear compositional change of microbiota after intervention in the group of SRP plus antibiotic, which could not be seen in the group receiving SRP plus placebo. 4. Within the group of SRP plus placebo, there was no significant difference in high‐abundant aRSVs after intervention, while within the group of SRP plus antibiotics, there was significant decrease in 10 high‐abundant aRSVs which belong to the complex associated with periodontitis.	SPR plus antibiotics was effective in reducing richness, decreasing periodontitis‐associated genera, and changing bacterial composition structure, while SPR plus placebo did not have such effects, but both interventions improved clinical outcomes significantly
6	Pre–post interventional study	2	SRP	NA	Subgingival plaque	Subgingival plaque samples from mesio‐buccal site of the first molar (four samples per subject, not pooled) collected at baseline and after treatment with sterile Gracey curettes	1. Richness: Chao1 2. Alpha diversity: Shannon index 3. Beta diversity: PCoA based on weighted UniFrac distance, hierarchical clustering 4. Relative abundance	1. Alpha diversity decreased, while richness increased significantly after treatment. 2. PCoA showed that pre‐interventional samples of the two subjects aggregated in one group, and were different from the postinterventional samples. The two postinterventional samples also differ from each other. 3. Relative abundance showed among the six most abundant bacteria phyla *Actinobacteria* and *Proteobacteria* increased significantly, while *Bacteroidetes*, *Spirochetes*, *and Fusobacteria* decreased significantly.	SRP was effective in decreasing alpha diversity, changing bacteria composition structure, and reducing relative abundance of dominant bacteria in subgingival plaque samples.
7	RCT	4 Test: 2 Control: 2	SRP + 500 mg amoxicillin + 400 mg metronidazole, three times daily for 7 days	SRP	Subgingival plaque	Samples collected with sterile paper point from four teeth with at least one pocket depth > 6 mm (pooled) at baseline and after interventions with one sterile paper point	1. Richness: ACE 2. Evenness: Pielou's index 3. Alpha diversity: Shannon index, Simpson index 4. Relative abundance: percentage of read counts	1. Alpha diversity assessed by Shannon and Simpson indices increased after intervention regardless of the intervention type (direct comparison, not based on statistical analysis). 2. Pielou's index showed that the abundance of OTUs was more equally distributed regardless of the intervention type. 3. Relative abundance showed that Treponema and Filifactor decreased significantly after intervention in the group with SRP plus antibiotics. The genera *Porphyromonas* and *Tannerella* decreased, while *Prevotella*, *Selenomonas*, *Streptococcus*, *Actinomyces*, and *Rothia* increased in both groups.	Intervention (SRP or SPR plus antibiotics) was effective in increasing alpha diversity, changing composition structure from Gram‐negative Bacteroidetes to Gram‐positive Firmicutes in both groups.
8	Pre–post interventional study	2	SRP + 500 mg amoxicillin + 500 mg metronidazole, three times daily for 8 days	NA	Subgingival plaque	Subgingival plaque samples collected from maxillary first molars (pooled) at baseline and 8 weeks after intervention with paper point	1. Cumulative % of total reads 2. Relative abundance	1. Cumulative % of total reads showed a few species dominate within each sample, with a high proportion of reads both before and after interventions. 2. Relative abundance showed that Gram‐positive (*Streptococcus*, *Rothia*, *Actinomyces*) and the Gram‐negative (*Veillonella*) commensals had great increase, while red complex bacterial species (*Porphyromonas*, *Treponema*, *Tanerella*) had great decrease after intervention.	Intervention (SPR plus antibiotics) was effective in changing composition structure from pathogenic species to commensals.
9	Pre–post interventional study	12	SRP	NA	Subgingival plaque	Subgingival plaque samples (four samples per subject) collected at baseline and 6 weeks after intervention with paper point	1. Richness: Chao1 2. Alpha diversity: Shannon index 3. Beta diversity: PCoA based on weighted UniFrac distance, cladogram 4. Relative abundance 5. Correlation network	1. Shannon index showed that there was no significant difference in alpha diversity 2. PCoA indicated there was difference in the bacterial composition between the pre‐ and post‐treatment groups. 3. Core microbiome (prevalence > 70% and relative mean abundance > 2%) at baseline and after intervention was different. 4. Network of OTU with prevalence > 50% showed that after intervention, correlation nodes in the pathogen component increased significantly, high‐connectivity nodes only belonged to the pathogen component rather than distributed in health and pathogen components, high‐connectivity nodes had relatively lower abundance compared to those in pre‐interventional samples.	SRP was effective in decreasing alpha diversity, changing bacteria composition structure, reducing relative abundance and prevalence of certain bacteria species, and also resulted in a less coordinated microbial community in subgingival plaque samples.
10	Interventional study with concurrent comparison group	36 with gingivitis, mild to moderate periodontitis, or severe periodontitis 4 healthy control	SRP	NA	Subgingival plaque	Samples collected from the two deepest periodontal pockets (pooled) at baseline and at least 6 weeks later with a periodontal scaler	1. Beta diversity: UniFrac‐based PCoA 2. Relative abundance	1. PCoA did not show clear separation between the pre‐ and postintervention groups. 2. Relative abundance showed that Fusobacterium was significantly correlated with pocket depth in all samples. The flora after interventions differed between individual subjects.	The species‐level analysis of certain species found that after intervention, the relative abundance of disease‐related and health‐related bacteria showed great individual differences and no clear trend could be observed.
11	Pre–post interventional study	12	SRP	NA	Subgingival plaque	Sample collected from two affected tooth (not pooled) with a sterile Gracey curette at baseline, and within 4 to 19 weeks after interventions	1. Alpha diversity: Shannon index 2. Beta diversity: PCoA based on weighted UniFrac distance, hierarchical clustering 3. Relative abundance: percentage of read counts 4. Correlation network 5. Functional pathway analysis	1. Alpha diversity significantly decreased after intervention. 2. PCoA showed that the microbial composition was significantly different between diseased state at baseline and resolved states after intervention. Heatmap and cluster analysis showed disease‐ and health‐associated taxa. 3. Relative abundance showed that eight bacteria genera significantly abundant belong to previous red complex, while four bacteria genera in yellow and purple complex were significantly abundant in resolved state. 4. Correlation network showed that in the diseased state, both disease‐ and health‐associated bacteria correlated more than in the resolved state. 5. Functional pathway analysis showed significant difference between diseased state and resolved state after intervention, including flagellar assembly and chemotaxis.	SRP was effective in decreasing alpha diversity, changing composition structure toward health‐associated bacteria genera, decreasing microbial correlation, and reducing disease‐associated functional pathway.
12	Pre–post interventional study	19	SRP	NA	Supragingival plaque and saliva	Samples collected at baseline and within two years later 1. Supragingival plaque was collected from all teeth in the side of the upper half‐jaw that contained most teeth (pooled) with sterile curettes. 2. Stimulated saliva samples were collected after asking subjects to chew paraffin wax.	1. Richness: ACE, Chao1 2. Alpha diversity: Shannon index 3. Beta diversity: PCoA based on unweighted UniFrac distance 4. Relative abundance: mean read counts	1. The microbial richness estimated by the Chao I and ACE, and alpha diversity assessed by the Shannon index were significantly lower in supragingival plaque samples after intervention, while no change in the above indices was observed in saliva samples after intervention. 2. PCoA showed the strong distinct clustering between plaque and saliva samples. The compositional difference following periodontal therapy was smaller than between the two bacterial communities. There was a more significant compositional change after intervention in supragingival plaque than in saliva sample. 3. Relative abundance showed that predominant genera *Fusobacterium* and *Kingerlla* significantly decreased after intervention in supragingival plaque samples, while no such change was observed in saliva samples.	SRP had greater impact in reducing richness, diversity, and changing composition structure in the supragingival plaque samples than saliva samples.

Note

Ref No.: 1. Belstrom et al., 2018; 2. Bizzarro et al., 2016; 3. Califf et al., 2017; 4. Chen et al., 2018; 5. Hagenfeld et al., 2018; 6. Han et al., 2017; 7. Junemann et al., 2012; 8. Laksmana et al., 2012; 9. Liu et al., 2018; 10. Schwarzberg et al., 2014; 11. Shi et al., 2015; 12. Yamanaka et al., 2012

### 3.2 Study subjects

The study subjects were generally healthy adults, and the age range mentioned in seven articles was from 18 to 73 years (Belstrom et al., 2018; Califf et al., 2017; Han et al., 2017; Liu et al., 2018; Schwarzberg et al., 2014; Shi et al., 2015; Yamanaka et al., 2012). Three studies mentioned the ratio of male to female subjects was one to one (Belstrom et al., 2018; Califf et al., 2017; Yamanaka et al., 2012), while one study included exclusively female subjects (Han et al., 2017). The types of periodontitis in the selected papers included chronic periodontitis (CP) (Belstrom et al., 2018; Bizzarro et al., 2016; Califf et al., 2017; Chen et al., 2018; Hagenfeld et al., 2018; Junemann et al., 2012; Schwarzberg et al., 2014; Shi et al., 2015; Yamanaka et al., 2012) and generalized aggressive periodontitis (AgP) (Han et al., 2017; Laksmana et al., 2012; Liu et al., 2018). The summary of the demographic profiles (age, ethnicity, history of smoking) and baseline periodontal status (baseline pocket depth) of the study subjects in each selected study was recorded (Table S3).

### 3.3 Study type

Of the 12 included papers, four were interventional studies with concurrent comparison group (Bizzarro et al., 2016; Califf et al., 2017; Hagenfeld et al., 2018; Junemann et al., 2012) (three were RCT (Bizzarro et al., 2016; Hagenfeld et al., 2018; Junemann et al., 2012)), and the remaining eight were pre–post interventional studies with single arm (Belstrom et al., 2018; Chen et al., 2018; Han et al., 2017; Laksmana et al., 2012; Liu et al., 2018; Schwarzberg et al., 2014; Shi et al., 2015; Yamanaka et al., 2012). The interventions in these papers included mechanical plaque removal (scaling root planning) (Belstrom et al., 2018; Chen et al., 2018; Han et al., 2017; Liu et al., 2018; Schwarzberg et al., 2014; Shi et al., 2015; Yamanaka et al., 2012)), use of antiseptics (rinse with 0.25% sodium hypochlorite for 30 s twice a week) (Califf et al., 2017), mechanical plaque removal combined with systemic administration of antibiotics (Hagenfeld et al., 2018; Junemann et al., 2012; Laksmana et al., 2012), and antiseptics combined with systemic administration of antibiotics (Bizzarro et al., 2016).

### 3.4 Sample collection

Samples collected in the reviewed studies included subgingival plaque (Belstrom et al., 2018; Bizzarro et al., 2016; Califf et al., 2017; Chen et al., 2018; Hagenfeld et al., 2018; Han et al., 2017; Junemann et al., 2012; Laksmana et al., 2012; Liu et al., 2018; Schwarzberg et al., 2014; Shi et al., 2015), supragingival plaque (Califf et al., 2017; Yamanaka et al., 2012), and saliva (Belstrom et al., 2018; Chen et al., 2018; Yamanaka et al., 2012). Dental plaque collected with curette or paper point was from posterior molars (Chen et al., 2018; Han et al., 2017; Laksmana et al., 2012; Liu et al., 2018), or tooth with deep pocket ≥6 mm/deepest pocket (Belstrom et al., 2018; Bizzarro et al., 2016; Califf et al., 2017; Hagenfeld et al., 2018; Junemann et al., 2012; Schwarzberg et al., 2014), or all teeth of a certain region (Yamanaka et al., 2012), or sites which were not specified (Shi et al., 2015). Dental plaque samples were either pooled or kept as separate samples. Eight studies used pooled samples (Belstrom et al., 2018; Bizzarro et al., 2016; Califf et al., 2017 (supragingival plaque), Hagenfeld et al., 2018, Junemann et al., 2012, Laksmana et al., 2012, Schwarzberg et al., 2014, Yamanaka et al., 2012), while four studies used non‐pooled samples (Chen et al., 2018; Han et al., 2017; Liu et al., 2018; Shi et al., 2015). Salivary samples can be further divided into chewing‐stimulated (bite on paraffin wax for 5 min) (Yamanaka et al., 2012) and unstimulated saliva (Chen et al., 2018). Samples were collected both at baseline and after intervention (once or at multiple time points). The postinterventional sampling time frame in the included study ranged from 2 weeks (Belstrom et al., 2018; Califf et al., 2017) to 25 months (Yamanaka et al., 2012) after the intervention.

### 3.5 Metagenomic analysis

Nine of the included studies started the metagenomic analysis with constructing operational taxonomic units (OTUs): clusters of reads which were grouped by less than a fixed sequence dissimilarity threshold (Belstrom et al., 2018; Bizzarro et al., 2016; Califf et al., 2017; Chen et al., 2018; Han et al., 2017; Junemann et al., 2012; Liu et al., 2018; Shi et al., 2015; Yamanaka et al., 2012). Only one study used ribosomal sequencing variants (RSVs) as the basis of metagenomic analysis (Hagenfeld et al., 2018).

### 3.6 Alpha diversity, richness, and evenness

When alpha diversity is used to assess richness and evenness within a certain sample, habitat, or ecosystem (within‐sample diversity), it does not only take into account how many species are present but also how evenly each species is distributed. In the included studies, Shannon index (Chen et al., 2018; Hagenfeld et al., 2018; Han et al., 2017; Junemann et al., 2012; Liu et al., 2018; Shi et al., 2015; Yamanaka et al., 2012), Simpson index (Chen et al., 2018; Junemann et al., 2012; Liu et al., 2018), and Faith phylogenetic diversity (Califf et al., 2017) were the indices used to assess alpha diversity. In total, six pre–post studies (Belstrom et al., 2018; Chen et al., 2018; Han et al., 2017; Liu et al., 2018; Shi et al., 2015; Yamanaka et al., 2012) and three RCTs (Bizzarro et al., 2016; Hagenfeld et al., 2018; Junemann et al., 2012) reported the change of alpha diversity.

For pre–post interventional studies with the intervention of SRP, the findings regarding the effectiveness of SRP on alpha diversity in dental plaque samples were not consistent: Alpha diversity was found to be lower compared to baseline in four studies (Belstrom et al., 2018 (two and six weeks after intervention), Han et al., 2017, Shi et al., 2015, Yamanaka et al., 2012), and similar in two studies (Chen et al., 2018; Liu et al., 2018). Among the above pre–post studies with the intervention of SRP, two studies reported the change of alpha diversity in saliva samples after intervention as well. The findings were also not consistent: One revealed a decrease in alpha diversity (Belstrom et al., 2018), while the other could not find such a reduction (Yamanaka et al., 2012). Of note, Belstrom et al. observed that although the alpha diversity in dental plaque samples was significantly lower at 2 weeks and 6 weeks after SRP, it bounced back to an even higher level than baseline after 12 weeks (Belstrom et al., 2018).

Two RCTs had the same design of intervention (SPR plus 500 mg amoxicillin and 400 mg metronidazole, three times daily for 7 days) and comparison groups (SRP) (Hagenfeld et al., 2018; Junemann et al., 2012). Within the interventional group of SRP plus antibiotics, the change of alpha diversity in dental plaque samples was not consistent: Alpha diversity was higher in one study (Junemann et al., 2012) while similar in the other study (Hagenfeld et al., 2018). Within the comparison group of SRP, the change of alpha diversity in dental plaque samples was also not consistent: Alpha diversity was higher in one study (Junemann et al., 2012) while similar in the other study (Hagenfeld et al., 2018). In the remaining RCT study, 0.12% chlorhexidine rinse plus antibiotics was implemented in the intervention group, while 0.12% chlorhexidine rinse was implemented in the comparison group (Bizzarro et al., 2016). No significant difference in alpha diversity was found within each group over time after baseline. In an interventional study with concurrent comparison group (randomization not mentioned), the effectiveness of the intervention on alpha diversity was not reported, but it found that alpha diversity (Faith's PD) could be used as an indicator of pocket depth (Califf et al., 2017).

ACE (Junemann et al., 2012; Yamanaka et al., 2012), Chao 1 (Califf et al., 2017; Han et al., 2017; Liu et al., 2018; Yamanaka et al., 2012), and the number of RSVs (Hagenfeld et al., 2018) were used to assess richness in the selected studies. Three pre–post studies (Han et al., 2017; Liu et al., 2018; Yamanaka et al., 2012) and one RCT (Hagenfeld et al., 2018) reported the change of richness after baseline. For pre–post interventional studies with the intervention of SRP, the findings regarding the effectiveness of SRP on richness in dental plaque samples were not consistent: Compared to baseline, richness was significantly lower in one study (Yamanaka et al., 2012), significantly higher in one study (Han et al., 2017), and similar in one study (Liu et al., 2018). The only RCT, which reported the change of richness, found that within the invention group of SRP plus antibiotics, richness decreased significantly compared to baseline, while within the comparison group of only SRP, there was no significant change in richness (Hagenfeld et al., 2018).

Pielou's index was used to assess evenness in two RCTs with the same design of intervention and comparison groups (Hagenfeld et al., 2018; Junemann et al., 2012). Between‐group analysis in one of the RCTs showed that there was no significant difference in evenness between the intervention and control groups after intervention (Hagenfeld et al., 2018). There was also no significant change within each group after the intervention (Hagenfeld et al., 2018). In the other RCT, no such between‐group analysis was made and Pielou's index showed that the abundance of OTUs was more equally distributed within each group after the intervention (Junemann et al., 2012).

### 3.7 Beta diversity

Beta diversity is a measure for comparing microbial composition between different samples, habitats, or ecosystems (between‐sample diversity), or detecting the overall shift in microbial community after a certain period of time. Among the included studies, eight studies used principal coordinates analysis (PCoA) to assess beta diversity (Califf et al., 2017; Chen et al., 2018; Hagenfeld et al., 2018; Han et al., 2017; Liu et al., 2018; Schwarzberg et al., 2014; Shi et al., 2015; Yamanaka et al., 2012) and one study used principal component analysis (PCA) (Bizzarro et al., 2016). Beta diversity matrix can be calculated in different ways: incorporating quantitative information of sequence abundance (e.g., Bray–Curtis (Bizzarro et al., 2016; Hagenfeld et al., 2018)) or incorporating information of phylogenetic distances between species (weighted UniFrac (Califf et al., 2017; Han et al., 2017; Liu et al., 2018; Shi et al., 2015) or unweighted UniFrac (Yamanaka et al., 2012)). The advantage of PCoA and PCA is that they reduce the dimensionality of microbiome data so that a low‐dimensional graphical plot could be generated where distance between each dot represents the dissimilarities between two samples.

For dental plaque samples, four pre–post studies with the intervention of SRP revealed a clear compositional change after interventions compared to baseline (Han et al., 2017; Liu et al., 2018; Shi et al., 2015; Yamanaka et al., 2012), while two pre–post studies did not find such a clear compositional separation after SRP (Chen et al., 2018; Schwarzberg et al., 2014). It is noteworthy that beta diversity of dental plaque sample was more significantly different between individuals in resolved status than that in diseased status (Han et al., 2017; Shi et al., 2015). Two pre–post studies with the intervention of SRP found that for saliva samples, there was no compositional separation between samples collected after intervention and at baseline (Chen et al., 2018; Yamanaka et al., 2012).

Two RCTs reported the effectiveness of intervention on beta diversity (Bizzarro et al., 2016; Hagenfeld et al., 2018). In one RCT, between‐group analysis showed there was a clear compositional separation between intervention (0.12% chlorhexidine rinse plus antibiotics) and control groups (0.12% chlorhexidine rinse) at the 3‐month follow‐up (Bizzarro et al., 2016). Within‐group analysis found that there was a clear compositional separation between samples at baseline and all follow‐ups within each group. In the other RCT, PCoA showed that there was a clear compositional change after intervention within the group of SRP plus antibiotic, which could not be seen within the group only receiving SRP (Hagenfeld et al., 2018).

### 3.8 Relative abundance

The dominant genera in pre‐ and postinterventional samples and the major change of relative abundance of some genera in the included studies are demonstrated in Table 2. Some studies used statistical approach to assess the change of relative abundance (Bizzarro et al., 2016; Hagenfeld et al., 2018; Liu et al., 2018; Schwarzberg et al., 2014; Shi et al., 2015; Yamanaka et al., 2012), while some studies made the descriptive conclusion as to which species increases or decreases (Belstrom et al., 2018; Chen et al., 2018; Han et al., 2017; Junemann et al., 2012; Laksmana et al., 2012).

**Table 2:** Summary of pre‐ and postabundant genera, and increased/decreased genera after intervention in selected studies

Ref no.	Measurement of Relative Abundance	Pre‐intervention abundant genera	Postintervention abundant genera	Postintervention increase	Postintervention decrease
1	Proportion of reads	*Prevotella*, *Streptococcus*, *Veillonella*, *Neisseria*, *Fusobacterium* (saliva) *Prevotella*, *Treponema*, *Porphyromonas*, *Fusobacterium* (subgingival plaque)	*Prevotella*, *Streptococcus*, *Veillonella*, *Neisseria*, *Fusobacterium* (saliva) *Rothia*, *Prevotella*, *Streptococcus*, *Actinomyces* (subgingival plaque)	*Streptococcus*, *Rothia*, *Actinomyces* (subgingival plaque) Minor impact on saliva samples	*Porphyromonas*, *Treponema* (subgingival plaque) Minor impact on saliva samples
2	Proportion of reads^a^ (ANOVA)	*Porphyromonas*, *Treponema*, *Fusobacterium*, *Filifactor* among those who had better response to interventions regardless of groups	*Actinomyces*, *Streptococcus*, *Veillonella*, *Neisseria*, *Haemophilus* among those who had better response to interventions regardless of groups	*Neisseria*, *Rothia*, *Capnocytophaga*, *Streptococcus* (both intervention and control groups) *Veillonella*, *Haemophilus* (only in intervention group) *Parvimonas*, *Actinomyces* (only in control group)	*Filifactor*, *Tannerella*, uncultured *Clostridiales family xiii incertae sedis*, *Porphyromonas*, *Treponema*, uncultured *Synergistaceae* (both intervention and control groups) *Paludibacter*, *Fusobacterium*, *Parvimonas* (only in intervention group)
3	NM	*Porphyromonas*, *Desulfovibrio*, SHD−231, *Treponema*, *Haemophilus*, *Acholeplasma*, TG5 Family: *Mogibacteriaceae* (unclassified genus), *Mycoplasma*, Order: ML615J−28 (unclassified genus), Family: *Leptotrichiaceae* (unclassified genus), Family: *Pasteurellaceae* (unclassified genus), Family: *Pasteurellaceae* (unclassified genus), *Eikenella*, *Desulfobulbus*, *Pseudoramibacter*, *Eubacterium*, *Methylobacterium*, *Mogibacterium*, Family: *Gemellaceae* (unclassified genus), *Scardovia*	*Desulfovibrio*, Order: RF39 (unclassified genus), Family: *Cardiobacteriaceae* (unclassified genus), Family: *Leptotrichiaceae* (unclassified genus), *Streptococcus*, Order: ML615J−28 (unclassified genus), Family: *Aerococcaceae* (unclassified genus), *Methanobrevibacter*, *Pedobacter*, Class: *Bacilli* (unclassified genus), BE24, *Butyrivibrio*, *Peptococcus*, Order: *Acidimicrobiales* (unclassified genus), Family: *Paraprevotellaceae* (unclassified genus), Family: *Enterobacteriaceae* (unclassified genus), *Rhizobium*, *Aerococcus*, *Filifactor*, *Slackia*	NM	NM
4	Proportion of reads	*Porphyromonas*, *Tannerella*, *Desulfobulbus*, *Eubacterium*, *Phocaeicola*, *Mogibacterium* (saliva) *Filifactor*, *Desulfobulbus*, *Eubacterium*, *Hallella*, *Porphyromonas*, *Phocaeicola*, and others (total 28 taxa overabundant in subgingival plaque)	NM	NM	Twenty‐four taxa of the overabundant taxa in subgingival plaque and five taxa of the overabundant taxa in saliva decreased after interventions
5	Mean relative read count^a^ (using negative binomial distribution to account for overdispersion)	*Fusobacterium*, *Porphyromonas*, *Tannerella*, *Fretibacterium* (both groups)	NM	Among high‐abundant RSVs *Streptococcus*, *Veillonella* (intervention group) Among high‐abundant RSVs no statistically different (control group)	Among high‐abundant RSVs *Porphyromonas*,* Tannerella*, *Treponema*, *Prevotella*, *Campylobacter*, *Fusobacterium*, *Parvimonas*, *Fretibacterium*, *Filifactor*, *Oceanivirga* (intervention group) Among high‐abundant RSVs no significant difference (control group)
6	Proportion of reads in each sample	*Sharpea*, *Moryella*, *Fusobacterium*, *Johnsonella*, *Peptococcus*, *Peptostreptococcus*, *Treponema*, *TG5*, *Desulfobulbus*, *Filifactor*, *Tannerella*, *Porphyromonas*, *Megamonas*, *Escherichia*, *Selenomonas*, *Dialister*, *Megasphaera*, *Prevotella*, *Leptotrichia*, *Hylemonella*, *Campylobacter*, *Bacteroides*, *Syntrophomonas*	*Kingella*, *Sphingopyxis*, *Lautropia*, *Capnocytophaga*, *Neisseria*, *Aggregatibacter*, *Corynebacterium*, *Actinomyces*, *Parascardovia*, *Veillonella*, *Rothia*, *Streptococcus*	*Actinobacteria*, *Proteobacteria* (phyla)	*Bacteroidetes*,* Spirochaetes*, *Fusobacteria* (phyla)
7	Proportion of reads	*Porphyromonas*, *Prevotella*,* Treponema*, *Fusobacterium*, *Tannerella* (both groups)	*Prevotella*, *Streptococcus*, *Fusobacterium* (both groups)	*Prevotella*, *Selenomonas*, *Streptococcus*, *Actinomyces*, *Rothia* (both groups)	*Treponema*,* Filifactor*, *Porphyromonas*, *Tannerella* (intervention group) *Porphyromonas*, *Tannerella* (control group)
8	*Proportion* of reads in each sample	*Fusobacterium*, *Porphyromonas*, *Prevotella, Synergistetes sp., Filifactor*, *Actinomyces*, *Treponema*	*Fusobacterium*, *Porphyromonas*, *Prevotella*, *Streptococcus*, *Veillonella,*	*Streptococcus*,* Rothia*, *Actinomyces*, *Veillonella*	*Fusobacterium*, *Porphyromonas*, *Treponema*, *Tannerella*
9	LEfSe LDA score^a^	*Porphyromonas*, *Treponema*, *Fretibacterium*	*Streptococcus*, *Lautropia*, *Haemophilus*, *Actinomyces*	*Lautropia*, *Actinomyces*, *Haemophilus*	*Treponema*, *Porphyromonas*, *Fretibacterium*
10	NM	NM	NM	*Streptococcus*	*Prevotella, Fusobacterium*
11	Proportion of reads^a^ (ANOVA)	*Porphyromonas*,* Treponem, Tannerella*, *Olsenella*, *Peptostreptococcus*, *Synergistes*,* Filifactor, Mycoplasma*	*Actinomyces*, *Streptococcus, Rothia*,* Bergeyella*	Health‐associated genera	Socransky's red complex members
12	Proportion of reads^a^ (paired *t* test)	*Streptococcus*, *Prevotella, Veillonella*, *Rothia*, *Neisseria* (saliva) *Streptococcus*, *Leptotrichia*, *Actinomyces*, *Rothia*, *Fusobacterium* (supragingival plaque)	*Streptococcus*, *Prevotella*, *Veillonella*, *Rothia*, *Actinomyces* (saliva) *Streptococcus*, *Leptotrichia*, *Actinomyces*, *Rothia*, *Corynebacterium* (supragingival plaque)	*Atopobium* (saliva) *Corynebacterium* (supragingival plaque)	*Granulicatella*,* Capnocytophag* (saliva) *Fusobacterium*, *Kingella* (supragingival plaque)

Abbreviation: NM, Not mentioned.

Ref No.: 1. Belstrom et al., 2018; 2. Bizzarro et al., 2016; 3. Califf et al., 2017; 4. Chen et al., 2018; 5. Hagenfeld et al., 2018; 6. Han et al., 2017; 7. Junemann et al., 2012; 8. Laksmana et al., 2012; 9. Liu et al., 2018; 10. Schwarzberg et al., 2014; 11. Shi et al., 2015; 12. Yamanaka et al., 2012

^a^ Using statistical approach to compare the change of relative abundance

Some of the included papers also employed heatmap with hierarchical clustering or cladogram to visually display distributive and quantitative change in microbial community (Chen et al., 2018; Han et al., 2017; Liu et al., 2018; Shi et al., 2015). This kind of visual display helps to identify the “core microbiome” and how these species are related in phylogenic tree. As shown in Table 2, the most commonly found dominant genera at baseline in the included studies were *Porphyromonas*, *Treponema*, *Fusobacterium*, *Tannerella*, *Prevotella*, and *Filifactor*, and they were replaced by *Streptococcus*, *Actinomyces*, *Rothia*, and *Villonella* after intervention.

### 3.9 Correlation network analysis

Correlation network indicates synergic or antagonistic interactions between genera. Among the included papers, four studies used network analysis to investigate coordinated interaction in microbial community (Bizzarro et al., 2016; Chen et al., 2018; Liu et al., 2018; Shi et al., 2015). Two papers showed that the number of connection nodes between microorganisms decreased significantly after intervention (Chen et al., 2018; Shi et al., 2015). One paper did correlation network of OUT with prevalence > 50% and found that after intervention, the number of correlation nodes increased significantly in the pathogen component, but the density of the network became lower compared to that of pre‐interventional network (Liu et al., 2018). Also, after intervention those high‐connectivity nodes only distributed in the pathogen component rather than in both health and pathogen components. In the study by Bizzarro et al., the subjects were divided into two subgroups, those who had relatively good clinical outcomes and those not (Bizzarro et al., 2016). Among patients who did not respond well to interventions clinically, the intervention did not change the topology of correlation network significantly. Among patients who responded well to interventions clinically, the topology of correlation network changed significantly, from a fully connected network of health‐associated genera and four relatively separated networks of disease‐associated genera at baseline to one network consisting of one subnetwork of health‐associated genera and one subnetwork of disease‐associated genera (Bizzarro et al., 2016). This indicates that well‐connected commensals at baseline could be a predictor for better clinical outcomes.

### 3.10 Functional pathway analysis

Only one study performed functional pathway analysis and reported that among the 90 functional bacterial pathways found in the microbiome, 24 were overexpressed at baseline (Shi et al., 2015). Among those overexpressed pathways, the most noteworthy ones are flagellar assembly and chemotaxis. These two pathways were significantly more abundant at baseline compared to after intervention. These two functional pathways favor flagellated motile microbial species to grow, colonize, and penetrate oral epithelial cells.

## 4 DISCUSSION

Previous studies showed that detection of specific pathogens by using targeted microbial techniques (DNA probes, microarrays) was poorly predictive of the prognosis of periodontitis. Therefore, analyzing entire disturbed microbial community rather than several putative pathogens might be the key in understanding periodontitis (Berezow & Darveau, 2011; Friedrich, 2008). NGS technique enables us to unveil the whole picture of microbial communities of different niches in oral cavity (Lazarevic et al., 2009). The papers included in this review all adopted the NGS technique, although at different levels. Most of the included papers adopted NGS technique at the 16S RNA level, while NGS technique in the papers of Shi et al. (2015) and Califf et al. (2017) were at the whole genome level, which could explore functional pathways as well.

By using the search strategy in this systematic review, a large number of papers were retrieved from three major biomedical databases, which yielded a very comprehensive search result. The small number of eligible papers reveals the challenge in real world to employ metagenomic approach in interventional studies among human subjects, from recruiting subjects, implementing interventions, obtaining microbial samples, controlling confounding factors, to conducting large‐scale metagenomic analysis. There is a huge variance between the selected studies in terms of the demographic characteristics of the study subjects, the baseline periodontal status, the types of interventions, and the length of the follow‐up, all of which collectively affect to the outcomes. Due to the heterogeneity of the aforementioned, it is not possible to conduct any quantitative analysis to summarize the results.

Alpha diversity consists of two aspects, richness and evenness. In a particular environment, richness measures the number of different species, while evenness measures how individual species are distributed. Shannon index, Simpson index, and Faith phylogenetic diversity each has its strength: Shannon index emphasizing more on rare species, Simpson index giving more weight to evenness, and Faith phylogenetic diversity incorporating phylogenetic information (Kim et al., 2017). Although it is intuitive to infer that periodontal interventions should increase alpha diversity as a more diverse community is associated with greater resilience and healthier status from the ecological point of view (Proulx et al., 2010), most of the included studies did not found such a trend of increasing alpha diversity after intervention. First, periodontal disease is caused by complex alteration of entire microbial community rather than a few dominant pathogens; therefore, the microbial community does not simply become more diverse after periodontal intervention. Secondly, in a healthier state after intervention, the number (richness) and the loads (abundance) of pathogenic species may decrease, while those of commensal species may increase. Considering that both pathogens and commensals change simultaneously, it would be difficult to determine the magnitude and direction of change in richness and evenness. Therefore, it is inconclusive as to how alpha diversity changes after intervention.

Compared to alpha diversity, beta diversity is more robust to noise introduced by PCR and sequencing errors (Ley et al., 2008). It is also more meaningful to assess beta diversity than alpha diversity in interventional studies as it can demonstrate whether periodontal interventions resulted in compositional rearrangement in microbial community, which likely contributed to the recovery of periodontal tissue. The findings of a clear separation in PCoA/PCA scatterplot between samples at baseline and after interventions from the majority pre–post studies with SRP confirm that non‐surgical periodontal therapy (SRP) is effective in disrupting biofilm in periodontal niche (Cobb, 2002). Beta diversity in the included studies also highlights additional interesting findings. The increased difference in beta diversity between individual samples after intervention suggests that healthy periodontal environment welcomes a variety of health‐associated microorganisms to co‐exist (Shi et al., 2015). In the reviewed studies, the beta diversity of saliva samples after intervention remained relatively stable and was significantly different from that of the plaque samples, indicating that salivary sample is not an ideal substitute for dental plaque because it cannot accurately reflect the dynamic change in periodontal niche (Yamanaka et al., 2012).

More detailed information regarding how much taxonomic change was after intervention in the included studies was provided by relative abundance at the genera level as well as visually in the heatmap and cladogram. It is not surprising to find that in most of the included studies, Gram‐negative genera *Porphyromonas*, *Treponema*, *Fusobacterium*, *Tannerella*, *Prevotella*, and *Filifactor* were predominantly enriched in samples at baseline. This is consistent with some landmark works, which show that “red and orange complexes” are considered to play leading roles in the development of periodontitis (Curtis, Zenobia, & Darveau, 2011; Darveau, Hajishengallis, & Curtis, 2012; Socransky et al., 1998). *Porphyromonas gingivalis* is considered as a key pathogen in periodontitis, altering the amount and composition of oral commensals (Hasturk et al., 2007; Kumar et al., 2006), manipulating complements and leukocytes (Liang et al., 2011), finally leading to destructive inflammation in periodontal tissue. *Fusobacterium* is considered to be a critical bacterium in forming biofilm as it bridges early colonizing species and late colonizers such as *Porphyromonas gingivalis* (Kolenbrander et al., 2006). *Prevotella* includes several well‐known periopathogens (*Preveotella nigrescens*, *Preveotella intermedia,* and *Prevotella melaninogenica*) (Socransky & Haffajee, 2005). The majority of studies also demonstrated that *Streptococcus*, *Actinomyces*, *Rothia*, and *Villonella* increased and became dominant in the samples after interventions, which were collectively known as commensals (Kolenbrander et al., 2006). Other than the findings in “core microbiome” before and after interventions, a plethora of microorganisms, which could not be detected by conventional culture or probes technique, were also found in the included studies (Hagenfeld et al., 2018; Liu et al., 2018; Shi et al., 2015). Although they were present at a low abundance, they collectively accounted for a larger proportion than the classical disease‐ and health‐associated genera. There are some limitations in the included studies that prevent further studying those low‐abundant microorganisms. First, the majority of the included studies sequenced 400–500 bp at the 16S rRNA level, which might not be able to produce a taxonomic resolution down to the species level. As a result, it is not always possible to determine the pathogenic potential of the low‐abundant genera. Also, correlation network and functional analyses focus only on high‐abundant species. How the low‐abundant microorganisms interact with one another as well as the “core microbiome” and function in the entire community under the effect of the intervention is a missing piece of the puzzle. It should be also noted that the due to the small sample size, some of the selected studies were only able to summarize descriptively the change of relative abundance. For those studies which employed statistical approach to detect the change of relative abundance, the mainstay of statistical methods is still conventional ANOVA and paired *t* test. Only one study accounted for overdispersion of the distribution of relative abundance (Hagenfeld et al., 2018), and only one study used LEfSe analysis (Liu et al., 2018). In order to make more accurate inference regarding the change of relative abundance, more sophisticated statistical methods should be employed in future studies.

Findings of the included studies show that the study interventions did not only influence subgingival microbial community in the taxonomic composition but also the interactions between microorganisms. A preliminary finding generated from the limited number of studies with network analysis is that the interventions can reduce either the numbers of nodes/links or the density of network. This indicates that the interactions in the microbial community (especially the disease‐associated component) can be disrupted by the interventions, shifting from a symbiotic state to a dysbiotic state. The metagenomic changes can be used as outcome measures in future studies to investigate the effectiveness of different treatment modalities for periodontitis. For instance, current clinical evidence is inconclusive as whether full‐mouth scaling within 24 hr is more effective compared to conventional quadrant SRP (Eberhard, Jepsen, Jervoe‐Storm, Needleman, & Worthington, 2015). In this situation, evaluating the metagenomic outcomes to compare the effectiveness between different treatment modalities seems more sensitive than clinical outcomes.

It is noteworthy that some of included studies adopted adjunctive use of systemic antibiotics. Systemic administration of antibiotic may be a double‐edged knife in the treatment of periodontitis. Antibiotics can suppress commensals and lead to a dysbiotic status. A good example of dysbiotic alteration of gut commensal microbiome following long‐term use of antibiotics is *Clostridium difficile* infection (Bartlett, 2017). Findings of this review do not show that adjunctive use of systemic antibiotics in interventions of SRP leads to an additional significant difference in metagenomic outcomes. Therefore, its standard application in the treatment of periodontitis may not be warranted.

## 5 CONCLUSION

Existing evidence from metagenomic studies depicts a complex change in microbiome after periodontal intervention. However, due to the heterogeneity of the methods and outcomes adopted, only descriptive and preliminary findings could be summarized in this review. As periodontitis is currently viewed as a multifactorial disease caused by the interaction between the dysbiosis of the entire subgingival microbial community and the host inflammatory response to it, future studies evaluating the effectiveness of periodontal treatment should shift from merely assessing clinical outcomes and several classical periopathogens to the metagenomic outcomes relating to the entire microbial community.

## CONFLICTS OF INTEREST

None to declare.

## AUTHOR CONTRIBUTIONS

**Ya Zhang:** Data curation; **Yinliang Qi:** Data curation; **Edward C. M. Lo:** Writing – review & editing; **Colman McGrath:** Methodology; **May Lei Mei:** Writing‐review & editing; **Ruoxi Dai:** Funding acquisition; Investigation; Supervision; Writing – original draft.

## Supporting information

Appendix S1

Table S1

Table S2

Table S3
