# Supplementary material for: Using next‐generation sequencing to detect oral microbiome change following periodontal interventions: A systematic review
Source: Oral Dis. 2020 May 26;27(5):1073–89. doi: 10.1111/odi.13405 (PMC8247266; doi:10.1111/odi.13405)
Supplement: Supplementary file 1 — Appendix S1 [file ODI-27-1073-s004.doc]

**Appendix S1. Search Strategy in three databases**

**Search Strategy in PubMed**

1. Tooth Diseases [MeSH]
2. Mouth Diseases [MeSH]
3. Oral Health [MeSH]
4. Dental caries [MeSH]
5. Root caries [MeSH]
6. DMF index [MeSH]
7. Dental caries susceptibility [MeSH]
8. Dental caries activity tests [MeSH]
9. Oral Hygiene [MeSH]
10. Gingival diseases [MeSH]
11. Gingivitis [MeSH]
12. Gingival pocket [MeSH]
13. Periodontal diseases [MeSH]
14. Periodontal attachment loss [MeSH]
15. Periodontal debridement [MeSH]
16. Periodontal index [MeSH]
17. Periodontal pocket [MeSH]
18. Periodontal abscess [MeSH]
19. “tooth decay”
20. “tooth demineralization”
21. “tooth decalcification”
22. “probing depth”
23. “bleeding on probing”
24. periodont*
25. “plaque score”
26. #1 OR #2 OR #3 OR #4 OR #5 OR #6 OR #7 OR #8 OR #9 OR #10 OR #11 OR #12 OR #13 OR #14 OR #15 OR #16 OR #17 OR #18 OR #19 OR #20 OR #21 OR #22 OR #23 OR #24 OR #25
27. Metagenomics [MeSH]
28. Metagenome [MeSH]
29. High-Throughput Nucleotide Sequencing [MeSH]
30. microbiota [MeSH]
31. Genes, Bacterial [MeSH]
32. metagenomics
33. “16s rDNA”
34. “16s rRNA”
35. Pyrosequencing
36. “next-generation sequencing”
37. “Illumina sequencing”
38. “Functional gene array”
39. “Oral microbiome”
40. Bacteria*
41. “Bacterial diversity”
42. “Bacterial community”
43. #27 OR #28 OR #29 OR #30 OR #31 OR #32 OR #33 OR #34 OR #35 OR #36 OR #37 OR #38 OR #39 OR #40 OR #41 OR #42
44. #26 AND #43

**Search Strategy in Embase**

1. tooth disease/
2. mouth disease/
3. dental caries/
4. DMF index/
5. mouth hygiene/
6. gingiva disease/
7. gingivitis/
8. periodontal disease/
9. dental debridement/
10. periodontal index/
11. periodontal pocket/
12. periodontal abscess/
13. tooth decay.mp
14. tooth demineralization.mp
15. tooth decalcification.mp
16. probing depth.mp
17. bleeding on probing.mp
18. periodont*.mp
19. plaque score.mp
20. #1 OR #2 OR #3 OR #4 OR #5 OR #6 OR #7 OR #8 OR #9 OR #10 OR #11 OR #12 OR #13 OR #14 OR #15 OR #16 OR #17 OR #18 OR #19
21. metagenomics/
22. metagenome/
23. high throughput sequencing/
24. microflora/
25. bacterial gene/
26. 16s rDNA.mp
27. 16s rRNA.mp
28. pyrosequencing.mp
29. next-generation sequencing.mp
30. illumina sequencing.mp
31. functional gene array.mp
32. oral microbiome.mp
33. bacteria*.mp
34. bacterial diversity.mp
35. bacterial community.mp
36. #21 OR #22 OR #23 OR #24 OR #25 OR #26 OR #27 OR #28 OR #29 OR #30 OR #31 OR #32 OR #33 OR #34 OR #35
37. #20 AND #36

**Search Strategy in Web of Science**

1. “tooth disease*”
2. “mouth disease*”
3. “oral health”
4. “dental caries”
5. “root caries”
6. “DMF index”
7. “dental caries susceptibility”
8. “dental caries activity tests”
9. “tooth decay”
10. “oral hygiene”
11. “gingiv*”
12. “periodont*”
13. “tooth demineralization”
14. “tooth decalcification”
15. “probing depth”
16. “bleeding on probing”
17. “plaque score”
18. #1 OR #2 OR #3 OR #4 OR #5 OR #6 OR #7 OR #8 OR #9 OR #10 OR #11 OR #12 OR #13 OR #14 OR #15 OR #16 OR #17
19. “metagenom*”
20. “high-throughput nucleotide sequencing”
21. “16s rDNA”
22. “16s rRNA”
23. “pyrosequencing”
24. “next-generation sequencing”
25. “Illumina sequencing”
26. “functional gene array”
27. “microbio*”
28. “bacteria*
29. “bacterial diversity”
30. “bacterial community”
31. #19 OR #20 OR #21 OR #22 OR #23 OR #24 OR #25 OR #26 OR #27 OR #28 OR #29 OR #30
32. #18 AND #31
