# Supplementary material for: Using next‐generation sequencing to detect oral microbiome change following periodontal interventions: A systematic review
Source: Oral Dis. 2020 May 26;27(5):1073–89. doi: 10.1111/odi.13405 (PMC8247266; doi:10.1111/odi.13405)
Supplement: Supplementary file 3 — Table S2 [file ODI-27-1073-s001.docx]

|  | **Junemann 2012** | **Yamanaka 2012** | **Shi 2015** | **Califf 2017** | **Han 2017** | **Belstrom 2017** | **Chen 2018** | **Hagenfeld 2018** | **Liu 2018** | **Laksmana 2012** | **Schwarzber 2014** | **Bizzarro 2015** |
| --- | --- | --- | --- | --- | --- | --- | --- | --- | --- | --- | --- | --- |
| Was the allocation sequence generated adequately? | Unclear | NA | NA | NA | NA | NA | NA | Unclear | NA | NA | NA | Unclear |
| Was the allocation of treatment adequately concealed? | Unclear | NA | NA | NA | NA | NA | NA | Unclear | NA | NA | NA | Unclear |
| Were participants analyzed within the groups they were originally assigned to? | Yes | NA | NA | Yes | NA | NA | NA | Yes | NA | NA | Yes | Yes |
| Did the study apply inclusion/exclusion criteria uniformly to all comparison groups? | Yes | NA | NA | Yes | NA | NA | NA | Yes | NA | NA | Yes | Yes |
| Were cases and controls selected appropriately? | Yes | NA | NA | Yes | NA | NA | NA | Yes | NA | NA | Yes | Yes |
| Did the strategy for recruiting participants into the study differ across study groups? | No | NA | NA | No | NA | NA | NA | No | NA | NA | No | No |
| Does the design or analysis control account for important confounding and modifying variables through matching, stratification, multivariable analysis, or other approaches? | Unclear | Unclear | Unclear | Unclear | Unclear | Unclear | Unclear | Unclear | Unclear | Unclear | Unclear | Unclear |
| Did researchers rule out any impact from a concurrent intervention or an unintended exposure that might bias results? | Unclear | Unclear | Unclear | Unclear | Unclear | Unclear | Unclear | Unclear | Unclear | Unclear | Unclear | Unclear |
| Did the study maintain fidelity to the intervention protocol? | Yes | Yes | Yes | Yes | Yes | Yes | Yes | Yes | Yes | Yes | Yes | Yes |
| If attrition was concern, were missing data handled appropriately? | Unclear | Unclear | Unclear | Unclear | Unclear | Unclear | Unclear | Unclear | Unclear | Unclear | Unclear | Unclear |
| In prospective studies, was the length of follow-up different between groups, or in case-control studies, was the time period between her intervention/exposure and outcome the same for cases and controls? | Yes | NA | NA | Yes | NA | NA | NA | Yes | NA | NA | Yes | Yes |
| Were the outcome assessors blinded to the intervention or exposure status of participants? | Yes | Unclear | Unclear | Unclear | Unclear | Unclear | Unclear | Yes | Unclear | Unclear | Unclear | Yes |
| Were interventions/exposures assessed/defined using valid and reliable measures, implemented consistently across all study participants? | Yes | Yes | Yes | Yes | Yes | Yes | Yes | Yes | Yes | Yes | Yes | Yes |
| Were outcome assessed/defined using valid and reliable measures, implemented consistently across all study participants? | Yes | Yes | Yes | Yes | Yes | Yes | Yes | Yes | Yes | Yes | Yes | Yes |
| Were confounding variables assessed using valid and reliable measures, implemented consistently across all study participants? | NA | NA | NA | NA | NA | NA | NA | NA | NA | NA | NA | NA |
| Were the potential outcomes prespecified by the researchers? Are all prespecified outcomes reported? | Unclear | Unclear | Unclear | Unclear | Unclear | Unclear | Unclear | Unclear | Unclear | Unclear | Unclear | Unclear |

NA: not applicable
