# Supplementary material for: Using next‐generation sequencing to detect oral microbiome change following periodontal interventions: A systematic review
Source: Oral Dis. 2020 May 26;27(5):1073–89. doi: 10.1111/odi.13405 (PMC8247266; doi:10.1111/odi.13405)
Supplement: Supplementary file 4 — Table S3 [file ODI-27-1073-s002.docx]

**Supplementary Table 3. Demographic profiles and baseline pocket depth of the study subjects**

| Ref No. | Age | % of non-smokers | Pockets depth | Ethnicity |
| --- | --- | --- | --- | --- |
| 1 | Mean: 63 yrs., Range: 47 - 75 yrs | 72% non-smokers | Mean: 3.4 mm | NA |
| 2 | NA | 44% non-smokers | Mean:4.6 mm | NA |
| 3 | Mean: 41 yrs | NA | Each patient exhibited at least four separate teeth with a pocket depth of >= 6 mm | NA |
| 4 | Mean: 52 yrs  Range: 25 - 79 yrs | NA | 5.8 ± 1.3 | NA |
| 5 | NA | 100% non-smokers | Percentage of pocket depth > 5 mm:  Antibiotics group: 20.9% ± 14.0%  Placebo group: 17.9% ± 12.0% | NA |
| 6 | Range: 27-29 yrs | 100% non-smokers | NA | NA |
| 7 | NA | 100% non-smokers | Percentage of pocket depth >= 6 mm: >= 38% | NA |
| 8 | NA | NA | NA | NA |
| 9 | Mean: 30.75 yrs  Range: 26 - 35 yrs | NA | 4.99 mm | Chinese |
| 10 | Range: 21 - 40 yrs | NA | NA | NA |
| 11 | Mean: 53 yrs  Range: 37 - 65 yrs | 100% non-smokers | NA | NA |
| 12 | Range: 35 - 73 ys | NA | Percentage of pocket depth > 4 mm: 21.4% - 85.8% | NA |

Ref No.: 1. Belstrom et al., 2018; 2. Bizzarro et al., 2016; 3. Califf et al., 2017; 4. Chen et al., 2018; 5. Hagenfeld et al., 2018; 6. Han et al., 2017; 7. Junemann et al., 2012; 8. Laksmana et al., 2012; 9. Liu et al., 2018; 10. Schwarzberg et al., 2014; 11. Shi et al., 2015; 12. Yamanaka et al., 2012
